# Maternal recall of birthweight and birth size in Entebbe, Uganda

**DOI:** 10.1111/j.1365-3156.2012.03091.x

**Published:** 2012-09-20

**Authors:** Swaib A Lule, Emily L Webb, Juliet Ndibazza, Margaret Nampijja, Lawrence Muhangi, Florence Akello, Muhammed Kakande, Robert Kizindo, Alison M Elliott

**Affiliations:** 1 Medical Research Council/Uganda Virus Research InstituteEntebbe, Uganda; 2 London School of Hygiene and Tropical MedicineLondon, UK; 3 Entebbe HospitalEntebbe, Uganda

**Keywords:** birthweight, reliability, validity, uganda

## Abstract

**Objectives:**

To assess the reliability of maternally recalled birthweight and size in Entebbe, Uganda.

**Methods:**

The study population comprised 404 mothers, who were participants in the Entebbe Mother and Baby Study (EMaBS). Mothers were recruited to EMaBS during antenatal care, maternal characteristics were recorded during pregnancy, and birthweight was recorded at delivery. Four to seven years after delivery, mothers were asked to recall the child’s birthweight and size. Their responses were compared with the birthweight recorded in the EMaBS database.

**Results:**

Of 404 interviewed mothers, 303 (75%) were able to give an estimate of birthweight and for 265 of these EMaBS data on recorded birthweights were available. Women who were educated and whose children had low birth order were more likely to be able to give an estimate: 37 (14%) recalled the exact recorded birthweight; a further 52 (20%) were accurate to within 0.1 kg of the recorded weight. On average, mothers overestimated birthweight by 0.06 kg (95% CI: 0.00–0.13 kg, *P* = 0.04). Recalled and recorded birthweights showed moderate agreement with an intraclass correlation coefficient of 0.64. Four hundered mothers gave an estimate of birth size: the sensitivity and specificity of recalled birth size for classifying low birthweight were 76% (95% CI: 50–93%) and 70% (95% CI: 65–75%), respectively.

**Conclusions:**

Mothers’ recall of birthweight was not precise but in absence of other data, recall of birthweight and size may have some value in epidemiological studies in these settings.

## Introduction

Birthweight is an important predictor of future growth patterns (Hindmarsh *et al.* 2008) and of mortality and morbidities later in life (Barker *et al.* 1989; Gofin *et al.* 2000; Godfrey & Barker 2001). It is also vital in assessment of population health status (Gofin *et al.* 2000). Records of birthweight are seldom available to researchers investigating disease aetiology in developing countries (Walton *et al.* 2000; Catov *et al.* 2006). Maternally recalled birthweight is often the only available source of birthweight information for use in retrospective epidemiological studies, and this may introduce information bias.

In developed countries, several studies have examined concordance between the birthweight recalled by the mother and the recorded birthweight and have shown that maternally recalled birthweight is a good proxy for recorded weight (Gofin *et al.* 2000; Walton *et al.* 2000; Tate *et al.* 2005; Van Gelder & Roeleveld 2011). However, in developing countries, there is limited information on accuracy of maternally reported birthweight and birth size. A study in Brazil reported that mothers accurately recalled birthweight 12 months after delivery but that this accuracy decreased with time after birth (Araújo *et al.* 2007). In Taiwan, mothers over reported birthweight even within a few months after delivery (Li *et al.* 2006). In Cameroon, maternal recall was very poor (Mbuagbaw & Gofin 2010), whereas in Kenya, mother’s recall of low birthweight (<2.5 kg) was very good (Mung’ala-Odera & Newton 2001). Given this variability, we have taken the opportunity provided by our birth cohort (the Entebbe Mother and Baby Study; EMaBS) to assess the reliability of maternally recalled birthweight and the validity of maternally recalled birth size and their determinants in Uganda.

## Methods

Between April 2003 and November 2005, the EMaBS birth cohort was established to investigate the effect of antihelminthic treatment during pregnancy on the offspring’s response to immunisation and on susceptibility to infectious diseases. Two thousand five hundred and seven women attending antenatal care at Entebbe hospital were enrolled into the trial. Full details of the trial design and procedures are described elsewhere (Elliott *et al.* 2007). Babies delivered in Entebbe Hospital were weighed immediately after birth using scales graduated in 0.1 kg units (Fazzini SRL, Vimodrone, Italy) and recorded to the nearest 0.1 kg. For babies delivered elsewhere, birthweight was recorded as it appeared on the child health card. Birthweight was available for 1964 of the 2345 live births in the cohort (Ndibazza *et al.* 2010). The children are currently being followed up, with regular visits to the clinic both for scheduled and illness visits.

From 21 September to 8 December 2010, we interviewed sequentially the mother of each child who attended the study clinic. Children were 4–7 years old at the time of interview. Mothers were asked whether they still possessed the child health card showing the birthweight record of the child. Without reference to the health card, mothers were asked to recall the birthweight of their child and to give a categorical estimate of the birth size of the child (small, normal or large). These data were linked with antenatal and delivery information from the EMaBS database, thus allowing for comparison of recalled and recorded birthweight.

Reliability of maternal recall of birthweight was assessed by calculating the mean difference between recalled birthweight and recorded birthweight and conducting a paired *t*-test. The intraclass correlation coefficient was calculated as a measure of the agreement between reported and recalled birthweight. Recorded birthweight was categorised into low birthweight (<2.5 kg), normal birthweight (2.5–4.0 kg) and large birthweight (>4.0 kg). Sensitivity and specificity of a mother’s perception of small birth size in detecting low birthweight babies and of a mother’s perception of large birth size in detecting large birthweight babies were calculated.

Logistic regression was used to examine factors associated with mother’s recall of birthweight. Two binary outcomes were considered: first, ability to recall any numerical estimate of birthweight; second, ability to recall birthweight to within 0.1 kg of the recorded weight. Explanatory factors considered were mother’s age, education and socio-economic status, child’s birth order, gender, recorded birthweight and the child’s age at the time of this study. Multivariable analysis was used to adjust for the possible confounding effect of factors that were crudely associated with the outcome.

## Results

Between 21 September and 8 December 2010, 404 mothers were interviewed. Mothers who were interviewed were on average slightly older, were less likely to be primigravidae and had attended more routine study visits, than the remaining mothers enrolled in the EMaBS cohort whose children did not attend the clinic during this study period. Their children were less likely to have been born at home. Of the 404 children whose mothers were interviewed, 204 (51%) were male and 200 (49%) were female, with a mean age of 5.7 years (range, 4.5–7.5 years). One hundred and ninety-seven (49%) had attended the clinic because of illness, and 207 (51%) had attended for a routine visit. The average age of mother at the time of delivery of the study baby was 25 years (range, 15–45 years), and 356 (88%) said they still had the child health card. Three hundred and three (75%) of the women were able to give an estimate of birthweight; of the remaining 101 women who were unable to give an estimate of birthweight, 11 had delivered at home, and thus, birthweight is unlikely to have been measured (although one woman who delivered at home did give an estimate of birthweight). Characteristics of those who recalled and did not recall birthweight are shown in Table 1. Women who gave an estimate for birthweight were more likely to be educated, and their children were more likely to be of low birth order. There was a crude association between younger maternal age and ability to give an estimate of birthweight, but maternal age and birth order were associated, and multivariable analyses suggested that the association between age and ability to give an estimate of birthweight was mediated through birth order (Table 1).

**Table 1:** Comparison of maternal and child characteristics between mothers who gave an estimate of birthweight and those who did not

Characteristic	Total mothers interviewed *N* = 404	Number (%) mothers who estimated birthweight	Crude OR (95% CI)	*P*-value	Adjusted OR (95% CI)*	*P*-value*
Mother’s age at birth of child (years)						
15–19	72	59 (82%)	1	0.004 [trend]	1	0.95
20–24	153	121 (79%)	0.83 (0.41–1.70)		1.46 (0.63–3.39)	
25–29	100	73 (73%)	0.60 (0.28–1.26)		1.50 (0.54–4.13)	
30+	79	50 (63%)	0.38 (0.18–0.81)		1.19 (0.37–3.79)	
Mother’s education†						
None	13	3 (23%)	0.12 (0.03–0.48)	<0.001	0.11 (0.03–0.44)	0.002
Primary	183	132 (72%)	1		1	
Secondary	169	138 (82%)	1.72 (1.04–2.85)		1.36 (0.80–2.31)	
Tertiary	38	29 (76%)	1.24 (0.55–2.81)		0.97 (0.41–2.30)	
Birth order						
1	87	76 (87%)	1	<0.001 [trend]	1	0.02 [trend]
2	103	80 (78%)	0.50 (0.23–1.10)		0.51 (0.21–1.22)	
3–4	131	98 (75%)	0.43 (0.20–0.91)		0.40 (0.15–1.05)	
≥5	83	49 (59%)	0.21 (0.10–0.45)		0.23 (0.07–0.74)	
Sex of child						
Male	204	149 (73%)	1	0.36		
Female	200	154 (77%)	1.24 (0.79–1.94)			
Age of child (years)						
4	84	62 (74%)	1	0.68		
5	175	133 (76%)	1.12 (0.62–2.04)			
6	119	91 (76%)	1.15 (0.61–2.20)			
7	26	17 (65%)	0.67 (0.26–1.72)			
Recorded birthweight (kg)‡						
<2.5	17	13 (76%)	1	0.94		
2.5–4	302	241 (80%)	1.22 (0.38–3.86)			
>4	14	11 (79%)	1.13 (0.21–6.17)			

* Multivariable model included mother’s age at birth of child, mother’s education and birth order, the adjusted estimate for mother’s age is interpreted as the independent effect of age that does not act through birth order, controlling for mother’s education.

† One missing value.

‡ Restricted to the 333 children for whom a record of birthweight was available.

**Figure 1:**
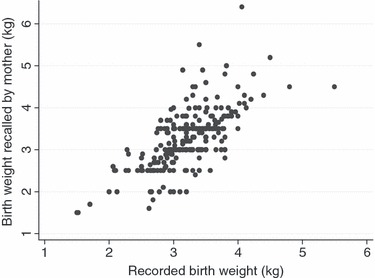
The relationship between recalled and recorded birth weights.

Analysis of the agreement between recorded and maternally recalled birthweight was restricted to 333 (82%) of the 404 interviewed mothers who gave birth in Entebbe hospital. Sixty-eight of these women were unable to recall their child’s birthweight, leaving 265 mother–child pairs with both a recalled and a recorded birthweight. The mean (standard deviation; range) of recalled and recorded birthweights were 3.28 kg (0.68 kg; 1.50–6.40 kg) and 3.21 kg (0.50 kg; 1.50–5.50 kg), respectively: on average, mothers overestimated the birthweight by 0.06 kg (95% CI: 0.00–0.13 kg, *P* = 0.04, paired *t*-test). Agreement between recalled and recorded birthweight was moderate (intraclass correlation coefficient 0.64, Figure 1). Only 37 (14%) of mothers recalled their child’s birthweight exactly as recorded; a further 52 (20%) recalled the birthweight to within 0.10 kg of the recorded value. Of the covariates considered, none was associated with accurate recall, or with the difference between recalled and recorded birthweight.

All but four of the 404 mothers gave a response regarding the size of the baby at birth. Thirty-five (9%) described their baby as large, 237 (59%) as normal and 128 (32%) as small. Reported size was associated with recorded birthweight (*P* < 0.001): the mean (SD) recorded birthweights for the recalled large, normal and small size groups were 3.73 kg (0.60 kg), 3.31 kg (0.40 kg) and 2.92 kg (0.49 kg), respectively. The sensitivity and specificity of mother’s recall of small size for low birthweight babies were 76% (95% CI: 50–93%) and 70% (95% CI: 65–75%), respectively, while the sensitivity and specificity for detecting large birthweight babies were 57% (29–82%) and 94% (91–97%), respectively.

## Discussion

This study from Uganda is one of a very small number of studies in sub-Saharan Africa to have assessed mothers’ recall of birthweight and birth size. Many mothers (25%) could not recall any numerical estimate of birthweight but almost all gave an approximate birth size. Those who gave an estimate of birthweight were not very accurate as to the precise figure, but there was moderate agreement between recalled and recorded birthweight. This was consistent with findings from the Netherlands by Jaspers *et al.* (2010) who found maternally recalled birthweight was not very accurate. Studies from the UK showed better maternal recall, with over 92% recalling birthweight to within 0.1 kg of recorded birthweight (Tate *et al.* 2005) and 85% to within 0.22 kg of recorded birth (Walton *et al.* 2000).

Studies by Rice *et al.* (2007), Gofin *et al.* (2000), O’Sullivan *et al.* (2000), Tate *et al.* (2005), Walton *et al.* (2000) and Jaspers *et al.* (2010) reported no mean difference between mothers’ recalled birthweight and recorded birthweight. In this study, we found there was a tendency of mothers to overestimate birthweight, and this finding was consistent with results from Taiwan reported by Li *et al.* (2006), but in contrast to findings from Denmark where mothers underestimated the birthweight (Adegboye & Heitmann 2008).

In this community, mothers’ concerns at birth are viability, absence of congenital anomalies and child’s sex and so little emphasis is placed on birthweight. Therefore, it is perhaps not surprising that many mothers in our study were unable to give a numerical estimate of weight. Ability to recall any numerical estimate of birthweight increased with education and decreased with the birth order of the child. However, we did not identify any maternal or child factors that were associated with accurate recall of birthweight. Similar results were reported by O’Sullivan *et al.* (2000), McCormick and Brooks-gunn (1999) and Olson *et al.* (1997). However, Tate *et al.* (2005) found that birth order, birthweight and socio-economic status influenced accurate recall of birthweight.

Mothers’ recall of birth size was more robust, allowing classification of babies as low birthweight with sensitivity and specificity of 76% and 70%, respectively (compared to a sensitivity of 60% and specificity of 93% seen in Cameroon (Mbuagbaw & Gofin 2010).

Possible sources of error and bias in this study were considered. Only one child per mother was enrolled into the EMaBS, and to be part of this analysis, the mother and child pair had attended the study clinic together; thus, it is unlikely that the mother gave data on any non-EMaBS sibling. Mothers who participated in this study were on average slightly older and had attended more routine study visits than members of the EMaBS cohort who were not included; and only 3% of their children had been born at home compared to EMaBS (Ndibazza *et al.* 2010) and community (Tann *et al.* 2007) estimates of 11%. Thus, some elements of our source population are under-represented. However, the differences in characteristics are not large; thus, any impact is likely to be minimal.

In summary, mother’s recall of birthweight was not precise but in absence of other data, mother’s recalled birthweight and birth size have some value for epidemiological studies, as long as it is not crucial to know the exact birthweight. Clinicians and researchers using maternally recalled birthweight should be cautious when using such information. Recalled birth size should be used only where recorded birthweight is not available.
